# Early vs late histological confirmation of coeliac disease in children with new-onset type 1 diabetes

**DOI:** 10.1007/s00125-022-05701-w

**Published:** 2022-04-30

**Authors:** Clemens Kamrath, Sascha R. Tittel, Desiree Dunstheimer, Elke Fröhlich-Reiterer, Markus Freff, Claudia Böttcher, Nadine Scheffler, Stefanie Lenze, Elke Gericke, Susanne Thiele, Reinhard W. Holl

**Affiliations:** 1 Division of Paediatric Endocrinology and Diabetology, Centre of Child and Adolescent Medicine, Justus Liebig University, Giessen, Germany; 2 Institute of Epidemiology and Medical Biometry (ZIBMT), Ulm University, Ulm, Germany; 3 German Center for Diabetes Research (DZD), Munich-Neuherberg, Germany; 4 Division of Paediatric Endocrinology and Diabetology, Centre of Child and Adolescent Medicine, University Children’s Hospital Augsburg, Augsburg, Germany; 5 Department of Paediatrics and Adolescent Medicine, Division of General Paediatrics, Medical University of Graz, Graz, Austria; 6 Darmstädter Kinderkliniken Prinzessin Margaret, Darmstadt, Germany; 7 Paediatric Endocrinology and Diabetology, University Children’s Hospital, University of Bern, Bern, Switzerland; 8 Centre for Paediatrics and Adolescent Medicine, Neonatology and Paediatric Intensive Care, Clinic Itzehoe, Itzehoe, Germany; 9 Centre of Diabetes, Department of Paediatrics, Sana Klinikum Berlin Lichtenberg, Berlin, Germany; 10 Centre of Child and Adolescent Medicine, Mathias-Spital Rheine, Rheine, Germany; 11 Centre of Diabetes, Hospital of Child and Adolescent Medicine, St Vincenz Hospital, Paderborn, Germany

**Keywords:** Autoimmune disease, Coeliac disease, Gluten, Polyendocrinopathy, Screening, Type 1 diabetes

## Abstract

**Aim:**

Screening for coeliac disease in asymptomatic children with new-onset type 1 diabetes is controversial. The aim of this study was to analyse whether the confirmation of coeliac disease in children with new-onset type 1 diabetes and positive screening results can be postponed.

**Methods:**

This was a multicentre population-based cohort study based on the German/Austrian/Swiss/Luxembourgian Prospective Diabetes Follow-up Registry (Diabetes Patienten Verlaufsdokumentation [DPV]). Participants aged ≤18 years diagnosed with type 1 diabetes between 1995 and June 2021 and with elevated IgA tissue transglutaminase antibodies (anti-tTGA) at diabetes onset on screening for coeliac disease were included. We compared outcomes of participants with a diabetes duration of more than 1 year between those in whom coeliac disease was confirmed histologically within the first 6 months and those in whom coeliac disease was confirmed between 6 and 36 months after diabetes diagnosis.

**Results:**

Of 92,278 children and adolescents with a diagnosis of type 1 diabetes, 26,952 (29.2%) had documented anti-tTGA data at diabetes onset. Of these, 2340 (8.7%) had an elevated anti-tTGA level. Individuals who screened positive were younger (median age 9.0 vs 9.8 years, *p*<0.001) and more often female (53.1% vs 44.4%, *p*<0.001). A total of 533 participants (22.8% of those who screened positive) had a documented biopsy, of whom 444 had documented histological confirmation of coeliac disease. Of 411 participants with biopsy-proven coeliac disease within the first 36 months of diabetes and follow-up data, histological confirmation was performed in 264 (64.2%) within the first 6 months and in 147 (35.8%) between 6 and 36 months after diabetes onset. At follow-up (median diabetes duration 5.3 years and 5.1 years, respectively), estimated median HbA_1c_ levels (62.8 mmol/mol vs 62.2 mmol/mol [7.9% vs 7.8%]), cardiovascular risk markers (lipids, rate of microalbuminuria, blood pressure), rates of acute diabetes complications (diabetic ketoacidosis, severe hypoglycaemia) and the proportions of participants reaching anti-tTGA levels within the normal range did not differ between groups. Participants with delayed histological confirmation of coeliac disease showed no negative effects on growth or weight gain during the observation period.

**Conclusions:**

Our study suggests that the histological confirmation of coeliac disease in asymptomatic individuals with new-onset type 1 diabetes could be postponed.

**Graphical abstract:**

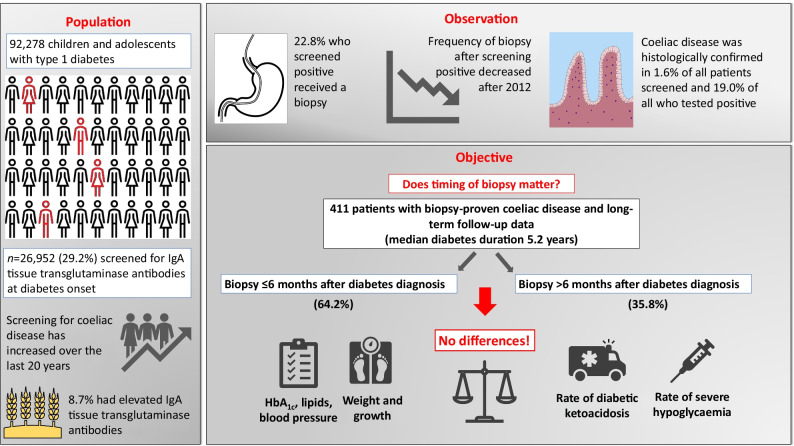

**Supplementary Information:**

The online version of this article (10.1007/s00125-022-05701-w) contains peer-reviewed but unedited supplementary material.

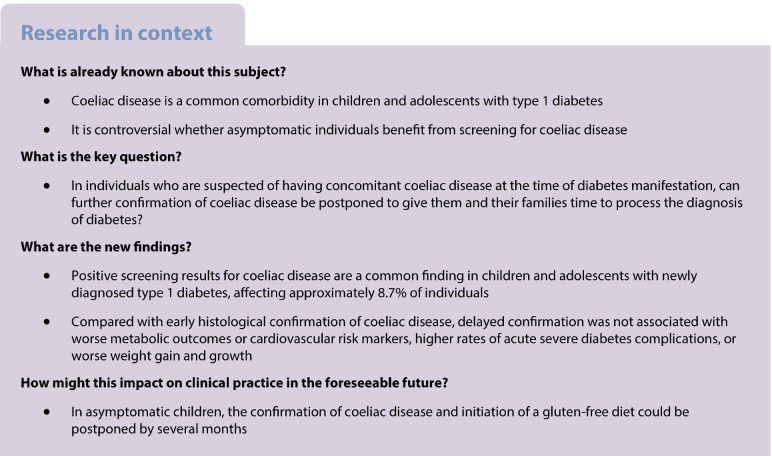



## Introduction

Coeliac disease is a common comorbidity in children and adolescents with type 1 diabetes [1–3]. Many individuals are asymptomatic at the time of diagnosis and are identified by screening for IgA antibodies against tissue transglutaminase (anti-tTGA) [4–6]. There is currently no clear evidence on whether asymptomatic individuals with type 1 diabetes benefit from a gluten-free diet (GFD) [7–10]. However, some studies have demonstrated that asymptomatic coeliac disease negatively affects growth and weight gain in children, although no effect on glycaemic control has been shown [11–13].

It has also been shown that mildly elevated serum anti-tTGA levels at diabetes onset decrease or even become negative over time in a significant proportion of children with type 1 diabetes, despite continued gluten consumption [14–16].

The aim of this study was to analyse if confirmation of coeliac disease in children and adolescents with new-onset type 1 diabetes and positive screening results can be postponed. We compared metabolic outcomes, cardiovascular risk factors, rates of acute complications, adherence to a GFD, and weight gain and growth of children and adolescents with new-onset type 1 diabetes and elevated anti-tTGA levels at diabetes onset who underwent early or late histological confirmation of coeliac disease.

## Methods

### Data source

This study is based on data from the German/Austrian/Swiss/Luxembourgian Prospective Diabetes Follow-up Registry (Diabetes Patienten Verlaufsdokumentation [DPV]) comprising 511 diabetes centres (hospitals and practices) and 630,352 individuals with diabetes as of June 2021. Twice a year, locally collected pseudonymised longitudinal data are transmitted for central plausibility checks and analyses to Ulm University, Ulm, Germany. Inconsistent data are reported back to participating centres for validation and/or correction. The data are then anonymised for benchmarking and patient-centred analyses [17].

### Study population

The study population included individuals up to the age of 18 years who received a diagnosis of type 1 diabetes between 1995 and June 2021 and who had elevated anti-tTGA titres above the upper limit of normal (ULN; according to the reference values of the respective laboratories of the treating centres) at diagnosis of diabetes (±11 days), with duodenal biopsy-proven coeliac disease within the first 36 months after diabetes onset. Biopsy-proven coeliac disease was defined as histopathology findings ≥ Marsh II [18]. We defined the biopsy date as the start of a GFD. Exclusion criteria were age <6 months or >18 years at the time of onset of type 1 diabetes, diabetes duration at last follow-up of <1 year, Marsh stage <II and lack of documented data at diabetes onset. We defined early biopsy-proven coeliac disease as biopsy within the first 6 months after diabetes onset, and delayed biopsy-proven coeliac disease as biopsy between 6 and 36 months after diagnosis of diabetes.

The data were analysed at onset of type 1 diabetes (±11 days), at the time of duodenal biopsy (±10 days), at follow-up 2 years after biopsy (±6 months) and at the most recent documented follow-up visit up to June 2021.

Applying the criteria described resulted in a study sample of 444 participants from 153 diabetes centres with biopsy-proven coeliac disease at diabetes onset, 411 participants with data at the most recent documented follow-up visit, 287 participants with documented data at the time of biopsy, and 367 participants with documented data 2 years after biopsy.

Verbal or written informed consent for participation in the DPV registry was obtained from patients or their guardians. The ethics committee of Ulm University approved the analysis of anonymized data from the DPV registry.

### Variables

The following demographic data were collected: age at diabetes onset, age at follow-up, sex, duration of diabetes, year of diabetes diagnosis, and immigrant background (participant or at least one parent born outside Germany/Austria/Switzerland/Luxembourg).

Anthropometric data were evaluated at onset of type 1 diabetes, at the time of duodenal biopsy, 2 years later and at the most recent follow-up visit. Data were collected on height (in centimetres) and BMI (calculated as weight in kilograms divided by height in metres squared). Height and BMI values were transformed into standard deviation score (SDS) values based on German reference values (German Health Interview and Examination Survey for Children and Adolescents [KiGGS]) by applying the Box Cox transformation method [19]. For the analysis of the BMI SDS values at the time of diabetes diagnosis, the weight at discharge from the inpatient stay was used [19]. However, we had documented data on weight and height at the time of biopsy for only 194 of 264 participants (73.5%) with early biopsy-proven coeliac disease and 83 of 147 participants (56.5%) with late biopsy-proven coeliac disease.

Clinical and metabolic outcomes were also evaluated at onset of type 1 diabetes, at the time of biopsy, 2 years later and at the most recent follow-up visit and included daily dose of insulin (units per kilogram body weight), use of real-time continuous glucose monitoring (CGM), use of an insulin pump (continuous subcutaneous insulin infusion), HbA_1c_ level (mmol/mol [%]), cardiovascular risk factors such as lipid levels (triacylglycerol, total cholesterol, HDL-cholesterol and LDL-cholesterol [all in mmol/l]), systolic blood pressure (mmHg; SDS), diastolic blood pressure (mmHg; SDS), rate of microalbuminuria (defined according to guidelines [20]) and rates of acute diabetes complications such as severe hypoglycaemia (with or without coma) and diabetic ketoacidosis (DKA). Systolic and diastolic blood pressure SDS values were calculated according to German reference values [21]. Severe hypoglycaemia was defined as an event with cognitive impairment (including coma and convulsions) requiring assistance from another person. Hypoglycaemic coma was defined as severe hypoglycaemia associated with seizure or loss of consciousness [22]. DKA was defined as pH <7.3 and/or serum bicarbonate <15 mmol/l [23].

In order to adjust for different laboratory methods, local HbA_1c_ values were mathematically standardised to the DCCT reference range (4.05–6.05%) using the ‘multiple of the mean’ transformation method [24].

### Statistical analyses

Unadjusted outcomes are presented as median (IQR) or percentage (%). Outcome data at follow-up were compared between individuals with early and individuals with delayed biopsy-proven coeliac disease using Wilcoxon’s rank sum test for continuous outcomes or the χ^2^ test for dichotomous outcomes.

Comparisons of the adjusted outcomes of HbA_1c_, daily dose of insulin and cardiovascular risk factors and of anthropometric data were analysed by linear regression and presented as estimated least-squares means with 95% CIs.

All models were adjusted for age at diabetes onset (as a continuous variable), sex, year of diabetes onset (as a continuous variable), diabetes duration (as a continuous variable) and immigrant background (participant or at least one parent born outside Germany, Austria, Switzerland or Luxembourg). Analysis of HbA_1c_ and daily dose of insulin were additionally adjusted for use of CGM or an insulin pump. Analysis of lipids and blood pressure were additionally adjusted for intake of lipid- or blood pressure-lowering drugs, respectively.

Rates of DKA and severe hypoglycaemia were estimated during the last year of follow-up using unadjusted negative binomial regression with individual time under risk as offset and are presented as rates per 100 person-years. Because of the low number of events, adjusted models did not converge in all analyses performed. In this case, frequencies of acute complications reported for the time interval since the last visit were analysed using an unadjusted logistic regression model.

A two-sided *p* value of ≤0.05 was considered statistically significant. All analyses were performed using SAS 9.4 (SAS Institute, Cary, NC, USA).

## Results

### Description of the study cohort

**Fig. 1:**
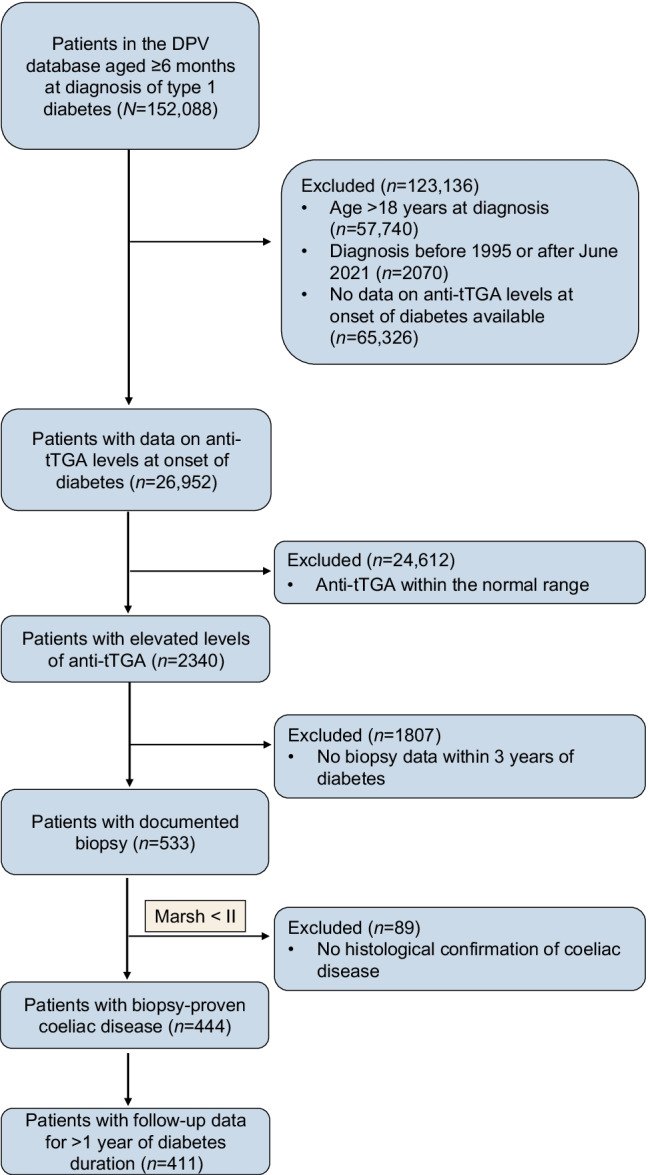
Selection of the study population. Inclusion criteria were diagnosis of type 1 diabetes between 1995 and June 2021, age between 6 months and 18 years at diagnosis, available baseline anti-tTGA data within 11 days of diabetes onset, elevated anti-tTGA titres above the ULN according to the reference values of the respective laboratories of the treating centres at diagnosis of diabetes, and biopsy-proven coeliac disease within the first 36 months of diabetes onset

**Fig. 2:**
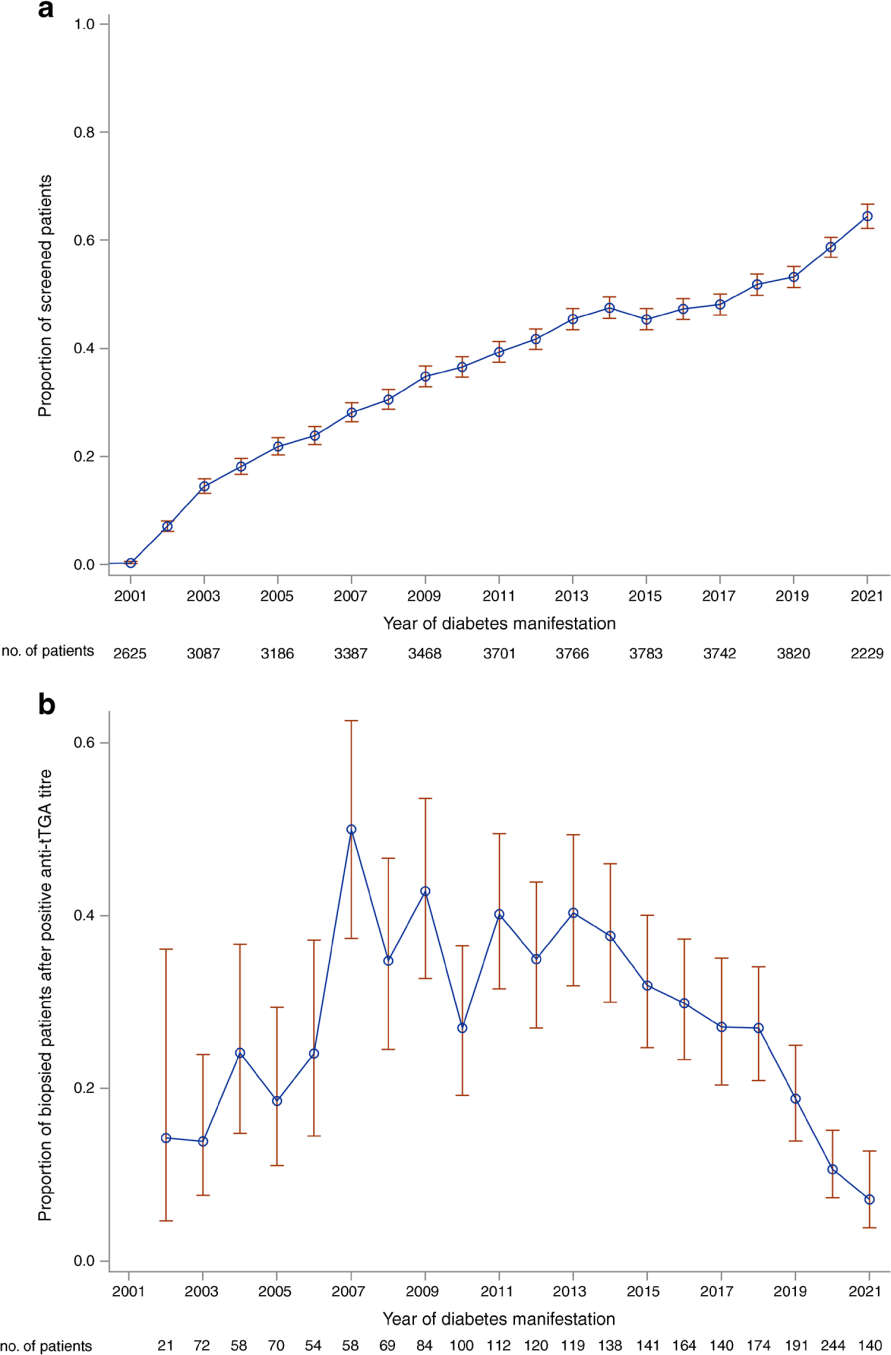
(**a**) Proportion of children and adolescents with new-onset type 1 diabetes screened for antibodies against tTGA at the onset of type 1 diabetes and (**b**) proportion who underwent histological examination for coeliac disease after a positive screening result from 2001 onwards. Error bars indicate 95% CIs

Of 92,278 children and adolescents in the DPV database with a diagnosis of type 1 diabetes between 1995 and June 2021 (of 152,088 individuals with type 1 diabetes in total), 26,952 (29.2%) had documented anti-tTGA data at diabetes onset (Fig. 1). The percentage of individuals with new-onset type 1 diabetes who were screened for coeliac disease increased from almost 0% in 2001 to over 60% in 2021 (Fig. 2a). Consequently, participants screened at onset of diabetes were diagnosed with type 1 diabetes later than those who were not screened (median year 2014 vs 2005, *p*<0.001).

Of the 26,952 children and adolescents with new-onset type 1 diabetes who were screened initially, 2340 (8.7%) had elevated anti-tTGA levels (Fig. 1). Compared with those with negative anti-tTGA results, individuals with elevated anti-tTGA levels were younger (median age 9.0 vs 9.8 years, *p*<0.001), were more often female (53.1% vs 44.4%, *p*<0.001) and more often had a concomitant diagnosis of autoimmune thyroiditis (Hashimoto’s thyroiditis: 8.7% vs 6.9%, *p*=0.003; Graves’ disease: 1.0% vs 0.5%; *p*=0.001) but not Addison’s disease (0.04% vs 0.06%, *p*=0.78).

Of these 2340 individuals with positive initial screening results for coeliac disease, 533 (22.8%) had a documented biopsy, of whom 444 (83.3% of those who received a biopsy, 19.0% of those who screened positive and 1.6% of all those screened for coeliac disease) had documented histological confirmation of coeliac disease (≥ Marsh II). In this group, anti-tTGA values > 10×ULN at onset of type 1 diabetes had a sensitivity of 80.6%, a specificity of 49.4%, a positive predictive value of 88.8%, a false-positive rate of 50.6% and a positive likelihood ratio of 1.6 for the histological confirmation of coeliac disease (see electronic supplementary material [ESM] Table 1).

An analysis of the frequency of biopsy in participants who screened positive showed an association between the year of type 1 diabetes onset and the frequency of performing a biopsy after abnormal screening (*p*<0.001). The percentage of participants undergoing a biopsy increased from well below 20% in 2002 to a plateau of about 40% from 2007 to 2013, followed by a decrease to about 20% in 2019 (Fig. 2b).

We obtained long-term data from 411 children and adolescents (170 male participants [41.4%]) with new-onset type 1 diabetes, initially elevated anti-tTGA levels and biopsy-proven coeliac disease within 36 months of diabetes. The median age of the entire cohort was 8.9 years (IQR 6.1–11.8) at diagnosis of type 1 diabetes and 15.8 years (IQR 11.9–17.4) at last follow-up. The median time between diagnosis of type 1 diabetes and histological confirmation of coeliac disease was 3.5 months (IQR 0.8–10.5). Median diabetes duration at follow-up was 5.2 years (IQR 3.1–7.7). An insulin pump was used by 230 participants (56.0%) and a CGM device was used by 255 participants (62.0%). Median HbA_1c_ during follow-up was 59.8 mmol/mol (IQR 52.7–70.5) (7.6% [IQR 7.0–8.6]). Documented anti-tTGA levels at last follow-up were available for 297 participants (72.3%). In total, 159 participants (53.5%) had anti-tTGA levels within the normal range at follow-up, while 53 participants (17.8%) had anti-tTGA levels > 10×ULN.

Of the 411 participants with biopsy-proven coeliac disease within 36 months of diabetes and available follow-up data, 264 (64.2%) underwent histological confirmation of coeliac disease early within the first 6 months after diabetes diagnosis (median diabetes duration at biopsy 1.3 months [IQR 0.5–3.3]), and 147 (35.8%) underwent biopsy later between 6 and 36 months after diabetes diagnosis (median diabetes duration at biopsy 13.5 months [IQR 9.1–18.9]). Median diabetes duration at follow-up was 5.3 years (IQR 2.8–7.9) in the group with early biopsy-proven coeliac disease and 5.1 years (IQR 3.4–7.2) in the group with delayed biopsy (*p*=1.00).

There were no differences between the groups in terms of sex, immigrant background and use of insulin pumps or CGM devices at follow-up. Table 1 provides the characteristics of the study cohort.

**Table 1:** Characteristics of participants with early vs delayed histological confirmation of coeliac disease and long-term follow-up data (*n =* 411)

Variable	Early biopsy (*n =* 264)	Delayed biopsy (*n =* 147)	*p* value
Age at diabetes diagnosis (years)	8.5 (5.8–11.3)	9.4 (6.2–12.4)	0.88
Duration of diabetes at biopsy (months)	1.3 (0.5–3.3)	13.5 (9.1–18.9)	
Duration of diabetes at last follow-up (years)	5.3 (2.8–7.9)	5.1 (3.4–7.2)	1.00
Age at last follow-up (years)	15.4 (11.8–17.3)	16.6 (12.4–17.4)	0.34
Female	149 (56.4)	92 (62.6)	1.00
DKA at diagnosis^a^	47 (18.7)	29 (20.9)	1.00
Immigrant background	56 (21.2)	26 (17.7)	1.00
Use of CGM at last follow-up	170 (64.4)	85 (57.8)	1.00
Use of insulin pump at last follow-up^b^	155 (58.7)	75 (51.4)	1.00
Anti-tTGA titre >10×ULN at diabetes diagnosis	224 (84.8)	108 (73.5)	0.06
Anti-tTGA titre <ULN at last follow-up^c^	105 (57.7)	54 (47.0)	0.64
Anti-tTGA titre >10×ULN at last follow-up^c^	32 (17.6)	21 (18.3)	1.00

Data are median (IQR) or *n* (%)

^a^Data on DKA at diagnosis were available for 252 participants in the early group and 139 participants in the late group

^b^Data on insulin pump usage at last follow-up were available for 146 participants in the late group

^c^Data on anti-tTGA levels at last follow-up were available for 182 participants in the early group and 115 participants in the late group

### Metabolic control and cardiovascular risk factors in participants with early vs late biopsy-proven coeliac disease

The adjusted mean HbA_1c_ levels during last follow-up did not differ between those with early biopsy-proven coeliac disease and those with late biopsy-proven coeliac disease (mean estimated HbA_1c_ 62.8 mmol/mol [95% CI 61.1, 64.5], 7.9% [95% CI 7.7, 8.0] vs 62.2 mmol/mol [95% CI 59.9, 64.5], 7.8% [95% CI 7.6, 8.1], *p=*0.71) (Table 2). Participants with postponed confirmation of coeliac disease had lower LDL-cholesterol levels 2 years after biopsy (ESM Table 2). However, during the last follow-up, the estimated daily dose of insulin, the cardiovascular risk markers total cholesterol, HDL-cholesterol, LDL-cholesterol, triacylglycerol and systolic and diastolic blood pressure, and rates of microalbuminuria did not differ between the groups (Table 2).

**Table 2:** Adjusted outcome variables at last follow-up for participants with early vs delayed histological confirmation of coeliac disease (*n* = 411)

Variable	Early biopsy (*n* = 264)	Delayed biopsy (*n* = 147)	*p* value
HbA_1c_ (*n*=410)			
mmol/mol	62.8 (61.1, 64.5)	62.2 (59.9, 64.5)	0.71
%	7.9 (7.7, 8.0)	7.8 (7.6, 8.1)	
Daily dose of insulin (U/kg) (*n* = 410)	0.88 (0.84, 0.92)	0.89 (0.84, 0.95)	0.79
Height SDS (*n* = 409)	0.09 (–0.03, 0.21)	0.01 (–0.15, 0.16)	0.39
BMI SDS (*n* = 409)	0.17 (0.07, 0.28)	0.33 (0.19, 0.47)	0.08
Total cholesterol (mmol/l) (*n* = 342)	4.51 (4.36, 4.66)	4.36 (4.16, 4.55)	0.29
HDL-cholesterol (mmol/l) (*n* = 324)	1.62 (1.57, 1.67)	1.59 (1.53, 1.64)	0.36
LDL-cholesterol (mmol/l) (*n* = 324)	2.50 (2.40, 2.61)	2.41 (2.27, 2.55)	0.30
Triacylglycerol (mmol/l) (*n* = 328)	1.24 (1.14, 1.34)	1.11 (0.98, 1.24)	0.12
Systolic blood pressure (mmHg) (*n* = 410)	119.9 (118.7, 121.0)	119.8 (118.2, 121.3)	0.88
Systolic blood pressure SDS (*n* = 410)	0.74 (0.62, 0.86)	0.79 (0.63, 0.95)	0.65
Diastolic blood pressure (mmHg) (*n* = 410)	70.5 (69.7, 71.4)	71.4 (70.3, 72.6)	0.25
Diastolic blood pressure SDS (*n* = 410)	0.39 (0.26, 0.51)	0.51 (0.35, 0.68)	0.24
Rate of microalbuminuria (%) (*n* = 284)	10.9 (7.0, 16.7)	7.5 (3.8, 14.1)	0.33

Data are mean (95% CI)

Data are adjusted for age, sex, year of diagnosis, duration of diabetes and immigrant background. Estimated mean HbA_1c_ and daily dose of insulin were additionally adjusted for use of CGM and insulin pump. Estimated mean BMI SDS was additionally adjusted for daily insulin requirements. Estimates of lipids and blood pressure were additionally adjusted for the intake of lipid- and blood pressure-lowering drugs, respectively

### Anthropometry in participants with early vs late biopsy-proven coeliac disease

**Fig. 3:**
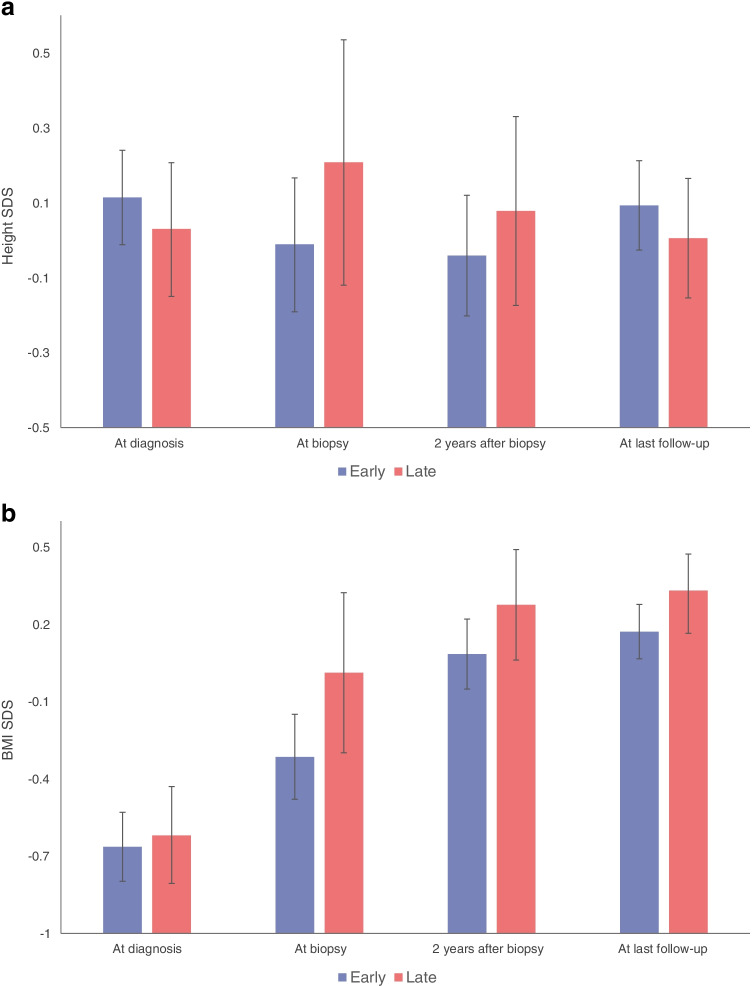
SDS values for (**a**) height and (**b**) BMI for participants with early vs delayed biopsy-proven coeliac disease. Error bars indicate 95% CIs. SDS values are shown at diagnosis of type 1 diabetes (*n* = 409 for both), at biopsy (*n* = 279 and 277, respectively), 2 years after biopsy (*n* = 365 for both) and at the last follow-up (*n*=409 for both; median diabetes duration 5.3 years and 5.1 years for the early and delayed biopsy groups, respectively)

Estimated mean height SDS and BMI SDS values did not differ between the early biopsy group and the late biopsy group at onset of diabetes, 2 years after biopsy and during the last follow-up (Fig. 3).

In addition, participants with later histological confirmation of coeliac disease showed no negative effects on growth or reduced weight gain during the observation period. Their estimated mean height SDS increased from 0.03 (95% CI –0.15, 0.21) to 0.21 (95% CI –0.12, 0.54) and their mean BMI SDS increased from –0.62 (95% CI –0.81, –0.43) to 0.01 (95% CI –0.30, 0.32) from onset of diabetes to the time of biopsy.

### Anti-tTGA levels and acute diabetes complications in participants with early vs late biopsy-proven coeliac disease

At diagnosis of type 1 diabetes, 224 of 264 participants (84.8%) with early histological confirmation of coeliac disease and available long-term follow-up data and 108 of 147 participants (73.5%) with delayed histological confirmation of coeliac disease had anti-TGA levels > 10×ULN (*p*=0.06; Table 1). At the last follow-up, neither the proportions of participants achieving anti-tTGA levels within the normal range (57.7% in the early group vs 47.0% in the late group, *p*=0.64) nor the proportions of participants with anti-tTGA levels > 10×ULN (17.6% in the early group vs 18.3% in the late group, *p*=1.00) differed between the two groups.

The rates or frequencies of acute diabetes complications such as severe hypoglycaemia (with or without coma) and DKA did not differ between the groups at the time of biopsy, 2 years after biopsy and at the last follow-up visit (Table 3).

**Table 3:** Estimated acute diabetes complications in participants with early vs delayed histological confirmation of coeliac disease (*n* = 411)

Complication	Early biopsy	Delayed biopsy	*p* value
Severe hypoglycaemia^a^			
At biopsy (*n* = 287)	12.7 (2.9, 55.4)	2.7 (0.1, 62.9)	0.38
2 years after biopsy (*n* = 367)	7.7 (4.2, 14.1)	12.5 (6.1, 25.7)	0.31
At last follow-up (*n* = 411)	8.5 (4.6, 15.5)	8.6 (3.8, 19.2)	0.98
Hypoglycaemic coma			
At biopsy (*n* = 287)	n.c.	n.c.	–
2 years after biopsy (*n* = 367)	n.c.	n.c.	–
At last follow-up (*n* = 411)	1.4 (0.4, 4.7)	2.4 (0.6, 9.3)	0.57
DKA^b^			
At biopsy (*n* = 287)	n.c.	n.c.	–
2 years after biopsy (*n* = 367)	0.8 (0.2, 3.4)	1.0 (0.2, 5.5)	0.81
At last follow-up (*n* = 411)	1.5 (0.6, 3.8)	1.5 (0.4, 5.1)	0.98

Data are number of acute diabetes complications per 100 person-years (95% CI)

Data were analysed at the time of biopsy (±10 days), at follow-up 2 years after biopsy (±6 months) and at the most recent documented follow-up visit up to June 2021

^a^Requiring assistance to treat hypoglycaemia

^b^pH < 7.3 and /or serum bicarbonate <15 mmol/l

n.c., not calculable (because of a lack of events)

## Discussion

This study found that elevated anti-tTGA levels were present in 8.7% of children and adolescents with new-onset type 1 diabetes screened for coeliac disease. Although screening for coeliac disease at diagnosis of type 1 diabetes is recommended in current and older guidelines [25–27], the proportion of participants screened in our evaluation was only 29%. However, there has been a clear trend toward more frequent screening for coeliac disease in children and adolescents with newly diagnosed type 1 diabetes over time, with a current rate of over 60%, showing that the guidelines are being implemented more frequently in routine care.

In addition, our evaluation shows that only 23% of individuals who screened positive underwent further histological clarification of coeliac disease. While there was an increase in biopsy frequency up to 2007, our data show a continuous decrease from 2013 onwards. This is most likely a consequence of the implementation of the 2012 guidelines of the European Society for Paediatric Gastroenterology, Hepatology and Nutrition, which allowed the serological diagnosis of coeliac disease without the need for histopathological examination [28]. However, this approach is in contrast to current guidelines on type 1 diabetes from the International Society for Pediatric and Adolescent Diabetes (ISPAD) and the American Diabetes Association, which continue to recommend a biopsy to confirm the diagnosis of coeliac disease in children and adolescents with type 1 diabetes [25, 26]. In addition, thresholds extrapolated from the general population for the diagnostic evaluation of coeliac disease are not thought to be appropriate for use in individuals with asymptomatic type 1 diabetes [29]. Consistent with this, our evaluation has shown that a single anti-TGA measurement >10×ULN at onset of type 1 diabetes is insufficient for an accurate diagnosis of coeliac disease.

Nevertheless, the number of individuals with histologically confirmed coeliac disease in the DPV registry is steadily increasing. While the proportion of individuals in the registry with biopsy-confirmed coeliac disease was 0.6% in 1995 and 1.3% in 2008 [13], it was 1.6% in this study.

In agreement with the current guidelines [25, 26], in those participants who underwent a biopsy for histological clarification, coeliac disease was confirmed soon after onset of type 1 diabetes in the majority of participants in our study. However, whether or not a GFD reduces the risk of complications in those with type 1 diabetes and coeliac disease remains to be investigated. It is important to note that studies investigating whether or not individuals with asymptomatic type 1 diabetes benefit from a GFD report inconsistent results, and the long-term benefits of a GFD in asymptomatic children identified by routine screening have not been proven [7–10]. Therefore, screening for coeliac disease in asymptomatic children with coexisting type 1 diabetes, which may already be a burden, is still controversial [30]. The additional burden of being diagnosed with a second autoimmune comorbidity close to the initial diagnosis of type 1 diabetes could have a negative psychosocial impact. For example, depression is twice as common in people with type 1 diabetes as in the general population [31], and the double load of type 1 diabetes and coeliac disease may lead to an increased risk of depression [32]. Unfortunately, however, our database analysis is unable to provide any information on possible psychosocial impacts. Carrying out an initial diagnostic assessment, on the other hand, may avoid the need for further hospitalisations. An elevated anti-tTGA finding without further clarification may also lead to uncertainty among patients and their families regarding their diet and consumption of gluten. In addition, deficiencies of micronutrients such as iron or zinc should be monitored in the case of a wait-and-see approach [33].

To date, no studies have investigated the difference between early and delayed confirmation of coeliac disease and initiation of a GFD in children with a concurrent diagnosis of type 1 diabetes and coeliac disease. In this study, delayed histological confirmation of coeliac disease was not associated with a worse long-term metabolic outcome or higher proportions of severe acute diabetes complications compared with early confirmation of coeliac disease. There was no difference in the cardiovascular risk profile between individuals with delayed histological confirmation of coeliac disease and initiation of a GFD and those with early confirmation. This is an important finding because it has been shown that individuals with type 1 diabetes and untreated coeliac disease have an unfavourable lipid profile that may increase their risk of cardiovascular disease [34, 35], and that coeliac disease is an independent risk factor for diabetes-related microvascular complications such as nephropathy in individuals with type 1 diabetes [36]. However, this risk was not associated with initiation of or adherence to a GFD [36].

It has been demonstrated that children with biopsy-proven coeliac disease have significantly lower weight and height SDS values than those without coeliac disease, which may result from delayed diagnosis and/ or inadequate adherence to a GFD [9–13]. We saw no differences in height SDS between individuals with delayed histological confirmation of coeliac disease, and thus delayed initiation of a GFD, and those undergoing an early biopsy. In addition, those undergoing a delayed biopsy showed adequate weight gain during the observation period. Furthermore, mean BMI SDS values did not differ between the groups during follow-up.

Normalisation of anti-tTGA titres is often used in routine clinical practice to estimate adherence to a GFD [37, 38]. Our study found no differences between the groups in the frequencies of participants who were able to achieve anti-tTGA values within the normal range. A later diagnosis therefore does not seem to have a negative impact on the acceptance of coeliac disease and a GFD.

It is important to note that serum anti-tTGA levels can spontaneously decrease or test results may even become negative in children with type 1 diabetes, despite gluten consumption [14–16]. Therefore, it has been suggested that, in asymptomatic individuals, histological confirmation of coeliac disease and initiation of a GFD should be delayed to avoid unnecessary interventions and reduce any additional psychological burden [14]. Our study suggests that confirmation of coeliac disease and initiation of a GFD in asymptomatic individuals with type 1 diabetes can be postponed. Therefore, the timing of further confirmation of coeliac disease in an individual with new-onset type 1 diabetes may be considered individually. Beside gastrointestinal symptoms, the family structure, burden of the diagnosis of type 1 diabetes and individual coping abilities should be taken into account.

The strengths of the present study include the large population-based sample size of more than 90,000 children and adolescents with type 1 diabetes, with stringent prospective data collection and a nationwide capture rate of more than 80% of the paediatric patients in Germany, Austria and Luxembourg [17]. This study provides data based on long-term real-world results from children with type 1 diabetes, with a median disease duration of approximately 5 years. Other studies that have analysed the effects of a GFD in asymptomatic individuals with type 1 diabetes and coeliac disease have included significantly fewer participants [7–10]. An additional strength of our study is that we included only individuals with histologically proven coeliac disease. To our knowledge, this is also the first study to compare outcomes between early and late histological confirmation of coeliac disease and initiation of a GFD in individuals who screened positive for coeliac disease at the onset of type 1 diabetes.

The limitations of our study include the lack of information about the individual indications for biopsy in each group. In addition, indications may differ between the diabetes centres. Another limitation of our study is that, of those with a positive screening result for coeliac disease but without a documented biopsy, the reason for this was recorded for only a minority of participants (50 participants did not undergo a biopsy because of a very high anti-tTGA titre and typical clinical signs of coeliac disease, and 12 participants refused the examination). The majority of participants lacked further documentation, and this group was significantly larger than the group that could be analysed. This will inevitably lead to a certain bias in the results. Furthermore, the impact of individual symptoms of coeliac disease could not be taken into account. We also have no data on iron or other micronutrient deficiencies. However, as the majority of patients with coeliac disease detected by screening are asymptomatic [4–6], and biopsies are performed late, we assumed that participants undergoing a late biopsy were asymptomatic.

In conclusion, our study demonstrates that positive screening results for coeliac disease are a common finding in children and adolescents with newly diagnosed type 1 diabetes. In asymptomatic children and adolescents with a positive screening result for coeliac disease, the confirmation of coeliac disease and initiation of a GFD may be delayed on an individual basis depending on the family structure, actual burden of the diagnosis of type 1 diabetes and individual coping abilities.

## Supplementary Information

ESM 1 (PDF 408 kb)

## Data Availability

The aggregated datasets of this study and the SAS code can be requested from RWH (e-mail: reinhard.holl@uni-ulm.de). Because of patient protection and patient consent, individual participant data will not be made available; however, remote data analysis is possible on approval of the DPV board.

## Abbreviations

CGM: Continuous glucose monitoring
DKA: Diabetic ketoacidosis
DPV: Diabetes Patienten Verlaufsdokumentation
GFD: Gluten-free diet
ISPAD: International Society for Pediatric and Adolescent Diabetes
SDS: Standard deviation score
tTGA: Tissue transglutaminase antibody
ULN: Upper limit of normal
