# Supplementary material for: Early vs late histological confirmation of coeliac disease in children with new-onset type 1 diabetes
Source: Diabetologia. 2022 Apr 30;65(7):1108–18. doi: 10.1007/s00125-022-05701-w (PMC9174128; doi:10.1007/s00125-022-05701-w)
Supplement: Supplementary file 1 — (PDF 408 kb) [file 125_2022_5701_MOESM1_ESM.pdf]

Electronic supplementary material

ESM Table 1. Test characteristics for anti IgA tissue transglutaminase-based screening at diagnosis of childhood type 1 diabetes for prediction of histological confirmation of celiac disease.

	**≥ Marsh II**	**< Marsh II**	**Total**
**anti-tTGA > 10 x ULN** – number (%)	358 (80.6)	45 (19.4)	403
**anti-tTGA >1 - ≤ 10 x ULN** – number (%)	86 (50.6)	44 (49.4)	130
**Total** – number	444	89	533

Test characteristics (95% CI) were: sensitivity 80.6% (76.6–84.2%); specificity 49.4% (38.7–60.3%); false positive rate 50.6% (39.8–61.3%); false negative rate 19.4% (15.8–23.4%); positive likelihood ratio 1.6 (1.3–2.0); negative likelihood ratio 0.4 (0.3–0.5); positive predictive value 88.8% (86.6–90.8%); negative predictive value 33.9% (27.8–40.4%); accuracy 75.4% (71.5–79.0%).

Abbreviations: anti-tTGA, anti-tissue transglutaminase IgA antibody; ULN, upper limit of normal.

ESM Table 2. Estimated[a] outcome parameters two years (+- 6 months) after the histological confirmation of celiac disease in patients with early vs. delayed small bowel biopsy.

**Variable** (number of patient's data for analysis)	**Early biopsy (N=238)** Mean (95% CI)	**Delayed biopsy (N=129)** Mean (95% CI)	**p-value**
**HbA_{1c}** – % [mmol/mol] (363)	7.6 (7.4–7.8) [59.7 (57.8–61.4)]	7.7 (7.5–7.9) [60.9 (58.4–63.4)]	0.44
**Daily dose of insulin –** U/kg (364)	0.79 (0.76–0.82)	0.83 (0.78–0.88)	0.24
**Cholesterol, total** – mmol/L (287)	4.47 (4.30–4.63)	4.32 (4.10–4.55)	0.32
**Cholesterol, high-density (HDL)** – mmol/L (262)	1.64 (1.59–1.70)	1.61 (1.53–1.68)	0.45
**Cholesterol, low-density (LDL)** – mmol/L (262)	2.48 (2.36–2.60)	2.25 (2.08–2.41)	0.028
**Triacylglycerol** – mg/dL (264)	1.09 (0.99–1.19)	1.09 (0.95–1.23)	>0.99
**Systolic blood pressure** – mmHg (361)	112.7 (111.5–113.9)	112.4 (110.8–114.8)	0.79
**Systolic blood pressure** – SDS (361)	0.72 (0.58–0.85)	0.58 (0.39–0.77)	0.26
**Diastolic blood pressure** – mmHg (361)	65.7 (64.8–66.7)	65.6 (65.2–67.9)	0.33
**Diastolic blood pressure** – SDS (361)	0.17 (0.03–0.31)	0.23 (0.04–0.42)	0.60
**Rate of microalbuminuria –** % (237)	9.9 (5.7–16.6)	5.2 (2.1–12.3)	0.19

Abbreviations: BMI-SDS, body mass index standard deviation score; HbA_{1c}, glycated hemoglobin; HDL, high-density lipoprotein; LDL, low-density lipoprotein.

[a] adjusted for age, sex, the year of diagnosis, duration of diabetes, and immigrant background. Estimated mean HbA_{1c} and daily dose of insulin were additionally adjusted to usage of CGM and insulin pump. Estimated mean BMI-SDS was additionally adjusted to daily insulin requirement. Estimates of lipids and blood pressure were additionally adjusted for the intake lipid- or blood pressure-lowering drugs, respectively.

Participating diabetes centers contributing data for the present analysis:
Ahlen St. Franziskus Kinderklinik, Aue Helios Kinderklink, Augsburg Josefinum Kinderklinik, Augsburg Uni-Kinderklinik, Aurich Kinderklinik, Bad Hersfeld Kinderklinik, Bad Kreuznach Diakonie Kinderklinik, Bad Kreuznach-Viktoriastift, Bad Kösen Median Kinderklinik, Bad Mergentheim - Diabetesfachklinik, Bad Orb Spessart Klinik, Bad Salzungen Kinderklinik, Berchtesgaden CJD, Berlin DRK-Kliniken Pädiatrie, Berlin Lichtenberg - Kinderklinik, Berlin Virchow-Kinderklinik, Bielefeld Kinderarztpraxis, Bielefeld Kinderklinik Gilead, Bochum Universitätskinderklinik St. Josef, Bonn Uni-Kinderklinik, Bremen - Kinderklinik Nord, Bremen Zentralkrankenhaus Kinderklinik, Bremerhaven Kinderklinik, Böblingen Kinderklinik, Chemnitz Kinderklinik, Coesfeld Kinderklinik, Darmstadt Kinderklinik Prinz. Margaret, Dornbirn Kinderklinik, Dortmund Kinderklinik, Dortmund Medizinische Kliniken Nord, Dresden Uni-Kinderklinik, Düren-Birkesdorf Kinderklinik, Düsseldorf Uni-Kinderklinik, Erfurt Kinderklinik, Erlangen Uni-Kinderklinik, Essen Elisabeth Kinderklinik, Feldkirch Kinderklinik, Filderstadt Kinderklinik, Freiburg Kinder-MVZ, Freiburg Uni-Kinderklinik, Garmisch-Partenkirchen Kinderklinik, Gelsenkirchen Kinderklinik Marienhospital, Gera Kinderklinik, Gießen Uni-Kinderklinik, Graz Uni-Kinderklinik, Greifswald Uni-Kinderklinik, Göttingen Uni Gastroenterologie, Göttingen Uni-Kinderklinik, Hagen Kinderklinik, Halle Uni-Kinderklinik, Hamburg Altonaer Kinderklinik, Hamburg Kinderklinik Wilhelmstift, Hameln Kinderklinik, Hanau Kinderklinik, Hannover Kinderklinik auf der Bult, Heidelberg Uni-Kinderklinik, Herdecke Kinderklinik, Herford Kinderarztpraxis, Heringsdorf Inselklinik, Hildesheim Bernward Krankenhaus Kinderheilkunde, Innsbruck Uni-Kinderklinik, Itzehoe Kinderklinik, Jena Kinderarztpraxis, Kaiserslautern-Westpfalzklinikum Kinderklinik, Karlsruhe Städtische Kinderklinik, Kassel Klinikum Kinder- und Jugendmedizin, Kiel Städtische Kinderklinik, Kiel Universitäts-Kinderklinik, Kirchen DRK Krankenhaus Kinderklinik, Koblenz Kinderklinik Kemperhof, Konstanz Kinderklinik, Krefeld Kinderklinik, Köln Kinderklinik Amsterdamerstrasse, Köln Uni-Kinderklinik, Landshut Kinderklink, Lappersdorf Kinderarztpraxis, Leverkusen Kinderklinik, Linz Krankenhaus der Barmherzigen Schwestern Kinderklinik, Lippstadt Evangelische Kinderklinik, Ludwigsburg Kinderklinik, Ludwigshafen Kinderklinik St.Anna-Stift, Luxembourg - Centre Hospitalier, Lübeck Uni-Kinderklinik, Lüdenscheid Märkische Kliniken - Kinder & Jugendmedizin, Magdeburg Kinderklinik St. Marienstift, Magdeburg Uni-Kinderklinik, Marburg Uni-Kinderklinik, Memmingen Kinderklinik, Minden Kinderklinik, Moers Kinderklinik, Mödling Kinderklinik, Mönchengladbach Kinderklinik Rheydt Elisabethkrankenhaus, München 3. Orden Kinderklinik, München von Haunersche Kinderklinik, München-Schwabing Kinderklinik, Münster St. Franziskus Kinderklinik, Nauen Havellandklinik, Neuburg Kinderklinik, Neunkirchen Marienhausklinik Kohlhof Kinderklinik, Neuss Lukas-Krankenhaus Kinderklinik, Neuwied Kinderklinik Elisabeth, Nürnberg Uniklinik Zentrum f Neugeb./Kinder & Jugendl., Oberhausen Kinderklinik, Oldenburg Schwerpunktpraxis Pädiatrie, Osnabrück Christliches Kinderhospital, Paderborn St. Vincenz Kinderklinik, Pforzheim Kinderklinik, Ravensburg Kinderklink St. Nikolaus, Regensburg Kinderklinik St. Hedwig, Rendsburg Kinderklinik, Reutlingen Kinderklinik, Rheine Mathiasspital Kinderklinik, Rosenheim Innere Medizin, Rosenheim Kinderklinik, Rotenburg/Wümme Agaplesion Diakonieklinikum Kinderabteilung, Saarbrücken Kinderklinik Winterberg, Salzburg Universitäts-Kinderklinik, Scheidegg Prinzregent

Luitpold, Schleswig Heliosklinik Kinderklinik, Schw. Gmünd Stauferklinik Kinderklinik, Schweinfurt Kinderklinik, Schwerin Kinderklinik, Siegen Kinderklinik, Singen Kinderarztpraxis, Speyer Diakonissen Stiftungskrankenhaus Pädiatrie, Stade Kinderklinik, Stolberg Kinderklinik, Stuttgart Olgahospital Kinderklinik, Sylt Rehaklinik, Trier Kinderklinik der Borromäerinnen, Vechta Kinderklinik, Vöcklabruck Kinderklinik, Waldshut Kinderpraxis, Wesel Marienhospital Kinderklinik, Wien 3. Med. Hietzing Innere, Wien KH Nord-Klinik Floridsdorf, Wien SMZ Ost Donauspital, Wien Uni-Kinderklinik, Wiesbaden Helios Horst-Schmidt-Kinderkliniken, Wiesbaden Kinderklinik DKD, Winnenden Rems-Murr Kinderklinik, Witten Kinderarztpraxis, Worms Kinderklinik, Wuppertal Universitäts-Kinderklinik.
